# Why we should not “help bad choosers:” screening, nudging, and epistemic risk

**DOI:** 10.1007/s11019-024-10217-8

**Published:** 2024-07-08

**Authors:** Jay Zameska

**Affiliations:** Interdisciplinary Centre for Ethics, Jagiellonian University, Grodzka 52, Krakow, 31-044 Poland

**Keywords:** Epistemic risk, Nudging, Ethics of screening, Patient decision-making, Healthcare ethics

## Abstract

One prominent line of support for nudging in screening programs is the claim that nudging can help ‘bad choosers’ — that is, it can help some patients make choices more in line with their own values and preferences. In this article, I argue that due to the presence of epistemic risk in many screening programs, the argument that nudging can help ‘bad choosers’ should be revised or rejected. Expanding on the work of Biddle, J. B. 2020. Epistemic risks in cancer screening: Implications for ethics and policy. *Studies in History and Philosophy of Science Part C: Studies in History and Philosophy of Biological and Biomedical Sciences* 79: 101200.), I argue that epistemic risk undermines the argument that nudging can help to promote patient autonomy in the context of screening. Specifically, I argue that epistemic risk results in the inclusion of non-patient values and preferences in the screening process, which challenges the claim that nudging can help patients make choices more in line with their own values and preferences. I present four reasons to think epistemic risk undermines the argument in this way: (1) conflicting values; (2) lack of transparency; (3) limited autonomy in opting out; (4) unjustified manipulation. The presence of epistemic risk in screening programs means that nudging may not always be an effective means of promoting patient autonomy and informed consent. As such, epistemic risk poses significant challenges to at least one ethical justification of nudging in screening programs, and raises further questions about the role of nudging in promoting patient decision-making.

## Introduction

Screening programs for a wide range of conditions enjoy significant support in high-income countries (Ebell et al. 2018; Olson et al. 2016). Screening is a strategy that involves preliminary testing or examination to detect signs of disease or abnormality, typically in asymptomatic individuals as part of a broader population survey.^1^ The most common aim of such programs is to improve population health through preventive measures and early intervention. As such, a major goal of many screening programs is to increase screening participation rates among the target population. One strategy that has become particularly prominent in recent years is the use of *nudging* to increase screening uptake (Hofmann and Stanak 2018).

Nudging involves “deliberate changes to and designs of people’s choice environments—the ways in which options are presented or framed—in attempts to predictably steer those people in specific directions” (Schmidt and Engelen 2020, p.2). Nudging has garnered significant academic and popular attention in a wide variety of contexts, and health care is no exception. Despite the enthusiasm that nudging has generated in public policy circles, there is still significant philosophical and ethical skepticism surrounding the practice (Kuyer and Gordijn 2023). However, a number of bioethicists have suggested that nudging may in fact help establish more robustly ethical screening programs. In particular, nudging has been suggested as a way to improve patient decision-making.

In this article, I address one kind of argument about nudging present in the bioethical literature on screening. Borrowing terminology from Hofmann and Stanak (2018), I call this kind of argument the “help bad choosers argument” or the HBCA. The basic idea of the HBCA is that nudging, when used appropriately, can help ‘bad choosers’ — that is, individuals who have some choice-related deficiency^2^ — make choices that are more in-line with their own values and preferences. For example, nudging has been suggested as a way to “raise the quality of [patient] decisions by reducing the extent to which they are subject to cognitive illusions and to which they make choices that they can be expected to regret” (Levy 2014, p.300). Given the importance of patient choice to both patient autonomy and informed consent, the HBCA seems to offer a strong reason to support certain kinds of nudging in screening programs.

Despite the promise of the HBCA, I believe its scope of application is significantly limited by the presence of *epistemic risk* in screening programs.^3^ Roughly put, epistemic risk is the risk of being wrong during knowledge-producing practices (Biddle 2020). It encompasses situations where there are clear and important risks beyond the risk of wrongly accepting or rejecting a hypothesis (known as “inductive risk”), and can include errors in disease definitions, endpoint measurements, and choices among models in screening programs.^4^ I will return to a more thorough discussion of epistemic risk later in this article.

Epistemic risk entails that there are substantive, non-epistemic values embedded in the screening process that cannot be entirely mitigated nor always communicated to the patient (Biddle 2020). This undermines the HBCA, as the presence of epistemic risk demonstrates that the HBCA fails to support its central claim: that nudging may promote patients’ abilities to make screening-related choices on the basis of their own values and preferences.^5^ As a result, epistemic risk weakens the HBCA as an ethical justification for nudging in screening programs. This does not necessarily mean that the HBCA is fatally flawed or never has application, but rather that the range of screening programs where the HBCA may be successfully applied is likely much more limited than previously assumed.

Before continuing, it is important to clarify that the focus of this article is not on the evidence for the effectiveness of screening programs or nudging. Even if there is excellent evidence that nudging successfully increases participation in a screening program, there may still be important ethical questions about whether nudging should be used. In such cases, evidence of effectiveness alone does not fully determine the ethical acceptability of the policy. A variety of other factors, including the notion of autonomy, also play a role in assessing the overall ethical status of a screening program that employs nudging. For example, screening programs that have good evidence of overall effectiveness still involve important value-laden considerations. Screening can lead to both undertreatment and overtreatment, depending on whether it yields false negative or false positive results in a given case. As I’ll discuss later, the presence of epistemic risk means that physicians and other decision-makers must make value judgments about which kinds of risk to prioritize, a question that is not fully determined by evidence of a program’s effectiveness. Furthermore, even when a screening program has an overall net benefit, it may still involve harm to some individuals that are “outweighed” by the aggregate benefits. For any given individual, there is a question of whether to take the gamble of being in the “benefited” or “harmed” category, and different moral frameworks may yield different judgments on this issue. A utilitarian perspective is likely to endorse programs with overall net benefits, while a Scanlonian contractualist approach would require justifying the program to each individual, including those who may be harmed. As such, my concern is with the justificatory structure of certain arguments for nudging, which turn on the role of patient values in justifying nudges, rather than the evidence of effectiveness for either nudging or screening.

In this article, I first introduce nudging and the “help bad choosers argument” (HBCA). Following this, I turn to the concept of epistemic risk, focusing on Biddle (2020)’s discussion of epistemic risk in cancer screening. I explain Biddles’ argument that epistemic risk is present in the diagnosis stage, and argue that it is also present in the *pre-appointment* or *pre-screening* stage. Next, I bring these elements together to argue that epistemic risk undermines the HBCA. In the penultimate section, I examine two possibilities to revise the HBCA, ultimately concluding that neither approach is entirely satisfactory. Finally, I conclude by indicating the limitations of this argument, its possible consequences, its significance to the bioethical literature, and noting some possible future directions of research.

## Nudging and the “help bad choosers argument”

In this section, I introduce nudging and the “help bad choosers argument” (HBCA), before moving to the next section to discuss epistemic risk. Nudging is based on the concept of *bounded rationality*. This is the idea that humans are not always fully rational in their decision-making, but are instead influenced by cognitive biases and heuristics due to limitations in cognitive abilities and processing capacity (see Kahneman, Slovic, and Tversky 1982; also Thaler and Sunstein 2009; Gigerenzer 2020).^6^ Nudging takes advantage of this element of human decision-making by structuring choices or presenting information in a way that guides people towards certain decisions, typically by making the desired option more salient, attractive, or easier, without entirely removing the ability to choose (Thaler and Sunstein 2009). It has been suggested as a means of helping patients make informed decisions that align with their values and goals. This includes, for example, influencing patient decisions as to whether to agree to surgery (Epstein 2017; Gorin et al. 2017), as well as encouraging people to participate in screening (Damhus et al. 2018; Hofmann and Stanak 2018).

Nudging has generated extensive discussion across a wide range of disciplines, including both health care and bioethics. There is considerable debate over whether and to what extent nudging is compatible with key concepts in health care ethics, particularly autonomy and informed consent. Although there is significant criticism of the ethical status of nudging in this regard (see, *inter alia*, Simkulet 2017; Nys and Engelen 2017; Waldron 2014; Hausman and Welch 2010; Coggon 2020), there is also significant support for the idea that nudging can promote both patient autonomy and informed consent (Brooks 2013; Douglas and Proudfoot 2013; Munoz et al. 2015).

Traditionally, healthcare providers have been encouraged to help facilitate patient autonomy by providing clear and understandable information, allowing sufficient time for decision-making, and offering support and guidance as needed, among other practices. Recently, however, there has been a trend towards employing nudging to promote patient autonomy. One particularly important strand of argument in favor of nudging in this context is what Hofmann and Stanak (2018) have labeled the “help bad choosers argument” for nudging (HBCA). The basic idea is that people are frequently ‘bad choosers’ — particularly in the context of screening — and may have difficulty making informed choices that are in-line with their own values and preferences. The HBCA holds that nudging may help patients to make decisions according to their own values and preferences, and thus, promote both patient autonomy and informed consent.^7^

The central idea is that because nudging in this area is intended to promote the patient’s ability to make choices on the basis of their own values and preferences, it is not only less ethically troubling than some other forms of nudging (e.g. those which aim to change patient values entirely),^8^ but it is actually autonomy-promoting, rather than autonomy-diminishing.^9^

There are a various versions of the HBCA in the literature, but here I focus on a generalized form:

*“Help bad choosers argument” (HBCA)*: Nudging, broadly defined, may enable patients to make “better choices” where “better” is understood to mean “more in line with the patient’s own values and preferences.

The HBCA appears in a variety of arguments in favor of various kinds of nudges in the health care literature, and is not limited to screening programs.^10^ However, for this article, I focus on the HBCA specifically and exclusively in the context of screening programs. This focus on screening programs is particularly relevant because there are significant concerns about patients’ and physicians’ abilities to understand the choices screening programs offer (see Hofmann 2020; Gigerenzer et al. 2007), which nudging is sometimes touted as able to improve or otherwise positively influence (see Blumenthal Barby and Burroughs 2012; Hofmann and Stanak 2018).^11^ As such, nudging in screening presents a particularly important area in which to analyze the HBCA.

However, I believe that the HBCA is flawed. The HBCA fails to take account of the presence of *epistemic risk* in screening, which allows for the entrance of non-patient values into the process of choice in a way that is not communicable to the patient (Biddle 2020). As such, it does not meet its own claim of helping patients to make decisions more in line with their own values. Instead, it opaquely includes others’ values and preferences. This means that the HBCA is not as ethically uncontroversial as it may first appear. Thus, epistemic risk in screening undermines the HBCA. To make this argument, however, first I need to introduce the concept of epistemic risk, which I do in the next section.

## Epistemic risk and screening

In this section, I introduce the concept of *epistemic risk*, with a particular focus on its role in medicine and health care. I then summarize an argument from Biddle (2020) that highlights the importance of epistemic risk to screening programs. Biddle argues that epistemic risk in screening makes it likely that “even fully rational patients might not have access to the information they need to make informed decisions” (2020, p.2). In the next section, I will expand on Biddle’s discussion, and argue that the presence of epistemic risk in screening undermines the HBCA.

It is worth nothing that Biddle (2020) already recognizes that his discussion is relevant to nudging. As he notes, his

paper raises significant challenges that autonomy-based approaches must overcome, if they are to succeed. If this is indeed the case, then it is an important result, particularly given that there are significant financial incentives for health care providers to nudge patients in the direction of treatment, even when treatment is unnecessary (Biddle 2016; Moynihan et al., 2012; Welch et al., 2011). Given these incentives, it is important to ensure that patients are not manipulated, under a guise of respect for autonomy, to undergo treatment that is against their interests. (Biddle 2020, p.2 citations in original)

However, Biddle does not explicitly address any of the arguments in favor of employing nudges in the context of screening, nor does he address the arguments that nudging may help to promote autonomy. Further, my focus here is not on financial incentives for physicians to nudge patients into treatment. Rather, my focus is on a certain class of bioethical arguments that claim to justify nudging, which are undermined by Biddle’s argument from epistemic risk. In this way, my focus is on a meta-bioethical level, which aims to show how epistemic risk undercuts a certain class of argument in the bioethical literature, rather than being directly applicable to the practice of health care as such.^12^ As a result, this work may be considered an extension of, or otherwise building on, the work of Biddle (2020).

In the simplest sense, “epistemic risk is the risk of being wrong.” (Biddle 2016 p.202). It can also be more broadly defined as “the risk of error that arises at any point in knowledge-productive practices (Biddle 2016, 2018; Biddle and Kukla 2017; Kukla, 2017).” (Biddle 2020, p.2, citations in original).^13^ Such risks may involve, for example, wrongly expanding (or narrowing) disease definitions (Biddle 2016), including or excluding borderline date points (Biddle and Kukla 2017; Biddle 2007), choices among models (Biddle and Kukla 2017), or the choice of “endpoint” or “outcome” measurements in cancer prevention (Plutynski 2017). The concept of epistemic risk was developed largely to address situations where the narrower concept of inductive risk — the risk of wrongly accepting or rejecting a hypothesis — did not directly apply, but where there were still clear and important risks.

However, recent work on epistemic risk has focused more specifically on epistemic risk in the context of medicine, and in particular, in the context of screening programs designed to promote public health. My focus here is Biddle (2020)’s work on epistemic risks in cancer screening. Biddle identifies and discusses three stages where epistemic risks that reflect substantive value judgements enter the process of prostate cancer diagnosis. The first stage is the selection of a threshold level for prostate-specific-antigen (PSA), which determines whether a biopsy is required. The second stage involves deciding how many samples to take in the event of a biopsy, and the third stage involves assigning a Gleason score to the biopsied samples (Biddle 2020). In each of these stages, Biddle demonstrates how there are epistemic risks — that is, risks of error or incorrect judgements — that introduce substantial value judgments.^14^

In his discussion, Biddle focuses on the *diagnosis stage* of screening: after an individual has already been screened, their test results must be analyzed, and epistemic risk arises during this process. However, I contend that epistemic risk is also present in the *pre-appointment* or *pre-screening* stage, before the patient actually undergoes any testing or examination. This is particularly relevant to the HBCA, because many nudges are intended to encourage screening uptake, and thus occur before any diagnostic procedures are carried out. Biddle’s discussion of epistemic risk in the diagnostic stage of screening is directly relevant to nudges that aim to, for example, encourage or discourage patients from seeking follow-up treatment on the basis of their test results. However, Biddle’s discussion of epistemic risk is less directly applicable to nudges that occur *before* the diagnostic stage, such as default scheduling of screening appointments, which constitute the majority of screening-related nudges (Hofmann and Stanak 2018). In the remainder of this section, I argue that epistemic risk is also present in the pre-appointment or pre-screening stage. I focus on two elements that introduce epistemic risk into the pre-screening stage. The first involves the definition of the disease to be screened for. The second involves the decision to seek out further health-related knowledge through screening.

First, disease definitions may introduce epistemic risk into the decision to participate in screening. The definition of a given disease may be more or less broad, which could result in increased false positives or false negatives. For example, the question of whether ductal carcinoma in situ (DCIS) should be properly categorized as a disease introduces epistemic risk (see Hoffmann 2016; Hofmann^2018^). If the disease definition is too broad or too narrow, there may be incorrect inclusion of individuals for further testing or incorrect exclusion of individuals who require further examination. Thus, epistemic risk occurs before patients enter into the screening program. As such, the presence of epistemic risk here is relevant to nudges that intend to increase participation rates.

Second, the decision whether to attend screening itself carries epistemic risk. Generally, the patient, or in the case of default scheduling, health policy makers, are predicting that such screening appointments will benefit the patient — something they may be wrong about, and something which requires substantive values to determine. Patients and other decision makers must weigh the potential benefits of early detection and treatment against the risks of unnecessary procedures, the potential for false positives and negatives, and the potential harm of overdiagnosis and overtreatment. Since such decisions are made under uncertainty, they include a degree of epistemic risk. As Biddle explains, “in most cases, when a judgment or decision is made in the face of uncertainty, the decision necessarily involves tradeoffs between different types of error, and the decision of which types of error one is more (or less) willing to tolerate reflects a set of values” (2020 p.2). As a result, the decision whether or not to participate in screening includes epistemic risk.

In this section, I’ve explained how epistemic risk in screening programs opaquely introduces non-patient values into the screening process in several ways. First, the decision to promote or encourage screening involves epistemic risk in the prediction that screening will benefit the patient. This prediction requires substantive value judgments by physicians or policymakers, who must weigh potential benefits against risks of harm. These value tradeoffs are not clearly communicated to patients. Second, disease definitions used in screening involve value judgments that are not transparent to patients. Value-laden decisions about how broadly or narrowly to define the screened-for disease can lead to more false positives or false negatives. Third, after the initial screening, deciding which PSA threshold requires a follow-up biopsy, or deciding how many biopsy samples to take, both introduce the values of physicians and lab technicians, as they must weigh risks of overdiagnosis against the risks of underdiagnosis. These values are not necessarily in alignment with the patient’s own and are not transparently conveyed. Finally, during diagnosis, the process of assigning Gleason scores to ambiguous biopsy results requires pathologists to make value-laden judgments, for example, deciding whether to err on the side of higher scores (and risk overdiagnosis) or on the side of lower scores (and risk underdiagnosis), in a way that is opaque to the patient.

In sum, epistemic risk is present in both the diagnosis stage and the pre-appointment stage of screening. As Biddle (2020) explains, diagnosis involves epistemic risk at multiple points in the process, ranging from setting a PSA threshold level to assigning Gleason scores. These value-laden judgments shape the options patients are presented with regarding follow-up testing and treatment. I argue the decision whether to attend screening also involves epistemic risk, as patients and/or policy makers assume that screening will benefit those screened, but they may be wrong, and substantive values are required to determine whether screening is appropriate. These values are directly relevant to patient decision-making about whether to participate in screening in the first place. In both cases, the various judgments required throughout the screening process, from defining diseases to assigning Gleason scores, introduce the values of physicians, technicians, and policymakers in a way that is not directly clear or communicable to patients, thus opaquely including others’ values that pertain to the decisions patients must make regarding screening participation, follow-up testing, and treatment into the process.

## Why nudging does not help ‘bad choosers’

As explained earlier, the centerpiece of the HBCA is the claim that nudging helps to promote the patient’s own values and preferences. As such, it is assumed to avoid the problems associated with nudges undermining autonomy or informed consent. For example, as Baldwin (2014) discusses,

“In further defence of the nudge it might be suggested that there is little need for debate when all the nudger is doing is […] trying to ‘influence choices in a way that will make choosers better off as judged by themselves’.” (Baldwin 2014, p.846. Baldwin quotes Thaler and Sunstein 2008, p.5.)

This quote captures the core idea of the HBCA: it is the patient’s *own* values and preferences that are relied on to make judgements about what is good for them and to determine what they should be nudged into. However, epistemic risk shows the HBCA to be inconsistent. It claims to promote patient’s welfare *by their own standards*, but this claim is undermined by the opaque and incommunicable inclusion of others’ values and preferences due to epistemic risk.

In short, the HBCA draws its justification from its claim that it promotes the patient’s own values and preferences. However, in this section, I argue that the presence of epistemic risk in the screening process undermines the HBCA. The HBCA does not only promote only the patient’s understanding of what is good for them, but it in fact also promotes the values and preferences of others, namely, the researchers, physicians, technicians, health policy makers, and others who must make decisions under epistemic risk at various stages of the screening process.

To review the discussion so far, the HBCA (“Help Bad Choosers Argument”) offers an ethical justification for nudging in the context of screening. The basic argument is that nudging can help individuals make ‘better’ choices, understood as choices that are more in line with their own values and preferences. However, due to epistemic risk, others’ values and preferences are included in various points of the screening process. The non-patient values and preferences introduced by epistemic risk warrant particular ethical concern because they may influence patient choice in the context of screening. This demarcates them from other instances of value inclusion that may be irrelevant to patient decision-making in this context. My claim is that this inclusion of others’ values and preferences undermines the HBCA, and thus, weakens the ethical justification for nudging in screening.

Why does the inclusion of others’ values and preferences undermine the justification for screening? I suggest four reasons: (1) conflicting values; (2) lack of transparency; (3) lack of understandable opt-out (4); unjustified manipulation. I will discuss each of these in turn.

First, the values and preferences of others may not be consistent with the values and preferences of the patient being nudged. People demonstrate a wide variety of attitudes and preferences around risk and risk management, with various levels of risk aversion and risk affinity. As such, there may be a mismatch between the risk-related preferences included in the screening process and the risk-related preferences of the patient. Thus, the nudge may not be promoting the individual’s own values and preferences, but a different and potentially incompatible set of values and preferences from unknown and unidentified others.

Second, when the values and preferences included in the nudges are not transparent or communicable, patients are unable to reflect on and deliberate about whether the nudges align with their own values and preferences. This lack of transparency and communicability undermines the prospective patient’s autonomy and decision-making ability, as they are not fully informed about the possibility for error. Similarly, they are also not fully informed about the choices made by others in light of this possibility for error. As Biddle (2020) demonstrates, the values and preferences included in the screening process are not easily or clearly communicable to patients (see pp.6–7). Thus, patients are unable to meaningfully agree to the inclusion of others’ values and preferences because they are unaware of the contents of their decision.^15^

Third, this inclusion of non-patient values and preferences undermines patients’ abilities to meaningfully opt out of screening appointments, follow up tests, or treatments after screening.^16^ Patients cannot opt out in a fully informed way because they do not know exactly what they are opting out of. Their decision is necessarily limited by their inability to know and understand the various values and preferences at play in the screening process. As a result, it is unclear whether either decision (opt in or opt out) really constitutes an informed decision. This is important because “opting out” of a nudge is often treated as a fail safe mechanism that is intended to preserve the “libertarian” elements of the “libertarian paternalism” that characterizes nudging (Sunstein and Thaler 2009, 5). However, due to epistemic risk, it is doubtful whether opting out can be considered to be a fully autonomous choice.

Fourth, the fact that other’s preferences are involved also raises concerns about the possibility of manipulation. Manipulation arises when a nudge “blocks the consideration of all options and threatens the agent’s ability to act in accordance with her or his own preferences (as opposed to someone else’s)” (Blumenthal-Barby and Burroughs 2012, p. 4). In the context of screening, epistemic risk may introduce such an instance of manipulation if the use of nudging blocks an agent’s ability to consider all options, and instead directs an agent to act in accordance with other’s preferences rather than their own. Although some hold that manipulation via nudge can sometimes be justifiable (see Nys and Engelen 2017), the problem is that it still undermines the stated goals of the HBCA to help patients make choices on the basis of *their own* values and preferences, rather than someone else’s. Thus, the HBCA would require further defense and support to justify such manipulation.

To clarify, the issue here is not about evidential uncertainty, but about the substantive value judgements introduced by epistemic risk. Epistemic risk involves making judgments under uncertainty that reflect tradeoffs between different types of error, and these judgments incorporate values - whether epistemic, social, ethical, or political - that influence the information and options presented to patients, undermining their ability to make autonomous choices aligned with their own values and preferences. Although general disclosures about epistemic or evidential uncertainty may be valuable, the core issue raised in the argument is that there are fundamental value judgments involved in the screening process that cannot be easily communicated to patients. This undermines the ability of nudges to truly promote patient autonomy, even if underlying evidential uncertainty is acknowledged and communicated. The problem I focus on here lies in the opacity of the value judgments, not just the evolving nature of medical knowledge. As such, much of this argument is contingent on the degree to which such values and preferences can be communicated to patients. If we could disclose such values, the force of the criticism of HBCA would be significantly lessened.^17^

## Revising the HBCA?

In light of the previous discussion, can the HBCA be revised to avoid the issues raised by epistemic risk? In this section, I address two possibilities for revising the HBCA in light of the challenge posed by epistemic risk. The first addresses the possibility of focusing on a population, rather than individual, version of the HBCA. The second attempts to attach the HBCA to a more robust ecological conception of autonomy.^18^ I briefly outline these possibilities, and argue that while these revisions may initially seem promising, they ultimately fail to provide a satisfactory solution to the problems raised by epistemic risk.

So far, I have focused primarily on analyzing the “help bad choosers argument” (HBCA) for nudging from an individual-level perspective, in line with the other work on the topic. One potential way to revise the HBCA in light of epistemic risk is to adopt a population-level perspective (I’ll call this the “Population-level HBCA,” or P-HBCA). Rather than focusing on that idea that nudges should promote each specific individual’s preferences, the P-HBCA would aim to improve overall health outcomes at the population level by considering the average or modal preferences of the population. This population approach could potentially mitigate some of the concerns raised by epistemic risk: focusing on group averages rather than specific individuals may attenuate the impact of any single instance of embedded values and preferences due to epistemic risk. If people on average share similar attitudes towards risk, for example, then the P-HBCA may do better than the original individual version.

However, the P-HBCA also faces significant ethical issues that undermine its ability to cohere with the motivating aims of the original HBCA. Shifting from the original HBCA to a “population-level HBCA” (P-HBCA) represents a fundamental change in the ethical framework and justification for using nudges. The original HBCA was grounded in promoting individual autonomy - the claim that nudges could help “bad choosers” make decisions more aligned with their own values and preferences. However, switching to the P-HBCA sacrifices the key focus on individual autonomy that grounded the original argument. It may provide a way around the challenges posed by epistemic risk, but it represents a major shift away from the HBCA’s original ethical framework and justification for nudging. The P-HBCA must rely on a different set of moral considerations, such as aiming to promote aggregate population benefits, rather than the original emphasis on autonomy and individual choice. Although the P-HBCA may avoid the specific problem posed by epistemic risk, it does so at the expense of abandoning the core ethical justification of the original HBCA - respect for individual autonomy.^19^

Overall, while modifying the HBCA into a P-HBCA may resolve the problem posed by epistemic risk, it fails as a satisfying solution because it abandons the fundamental value of individual autonomy that originally motivated and justified the HBCA. In short, the P-HBCA is a fundamentally different ethical argument, one no longer grounded in the respect for individual autonomy that was central to the original HBCA. Grounding nudges in population-level considerations is a significantly different ethical approach than the original claim of helping individuals make decisions aligned with their values and preferences. This leads the HBCA to a dilemma: either an individual-level framing encounters the challenges of epistemic risk, or a population approach avoids these challenges at the expense of discarding the core principle of promoting personal autonomy that defines and motivates the HBCA. As such, restructuring the HBCA to focus on populations may provide a stronger justification for nudging in screening, but it ultimately requires abandoning the focus on autonomy that defines and motivates the HBCA.

Given the limitations of the population approach, it is worth considering an alternative approach to revising the HBCA that aims to maintain its focus on individual autonomy by incorporating a more flexible, ecological conception of autonomy. Next, I evaluate the prospects for revising the HBCA to explicitly include an “ecological” conception of autonomy, which may be better suited to addressing the inclusion of others’ values and preferences due to epistemic risk.

In the remainder of this section, I attempt to present the strongest version of the HBCA by incorporating an “ecological” conception of autonomy based on Schmidt’s (2019) rationality-focused account. This ecological view holds that individuals exercise autonomy by drawing upon their specific environment and cognitive capabilities to reliably achieve their ends, rather than through strictly adhering to abstract norms of rational choice theory (Schmidt 2019, 521–522). This allows for a wider variety of exogenous influences, potentially including non-patient values and preferences, without undermining autonomy.

Under an ecological rationality view, the procedures people use to make choices can be considered rational insofar as they allow the individual to successfully navigate their circumstances and attain their desired ends, even if those procedures deviate from traditional rational choice theory (Schmidt 2019). Central to this view is the distinction between content rationality, which determines a ranking of ends based on an agent’s values and preferences, and procedural rationality, which concerns the decision-making procedures an agent uses to arrive at a choice. Ecological rationality focuses on procedural rationality while bracketing out questions of content rationality as a separate normative question (Schmidt 2019, p. 521).

Building on this view of rationality, an ecological autonomy account suggests that autonomous choice does not require being entirely free from external influences. Choices can be shaped by external factors in the choice architecture, as long as those factors ultimately help the chooser stay attuned to their own values and preferences when deciding which ends to pursue. This may allow such a view to rescue the HBCA: non-patient values and preferences are not necessarily autonomy-undermining, so long as nudging still helps patients track and adhere to their own ends, as defined by their own values and preferences. As long as it does not undermine the patient’s content rationality, such influences are not autonomy-undermining, and may even be autonomy-promoting in line with the HBCA.

However, I contend that even under this more flexible ecological conception of autonomy, epistemic risk still significantly undermines the core premise of the HBCA in screening contexts. Although nudges may influence procedural rationality, the non-patient values included through epistemic risk can directly conflict with and undermine a patient’s ability to set their own ends. In other words, it may undermine their content rationality. To reuse an example discussed above, epistemic risk arises when setting thresholds for further testing (e.g. PSA levels for prostate biopsies). The choice of threshold reflects value judgments about the relative costs of different types of errors (false positives vs. false negatives). These judgments can vary based on the pathologist’s own values and may conflict with the patient’s own preferences regarding the risks of different types of error (Biddle 2020).

These value-laden decisions made under epistemic risk end up shaping the outcomes or options that patients are presented with and asked to choose between. These non-patient values are opaquely included in the information provided to patients in a way that is difficult for them to identify and navigate (Biddle 2020). Rather than merely helping patients stay attuned to their own preferences, nudges in screening may steer patients toward choices misaligned with those preferences. This undermines a core requirement of both ecological autonomy and the HBCA.

In sum, even with a more flexible ecological conception of autonomy, epistemic risk still undermines the HBCA. Although an ecological view provides a plausible account of how nudges could promote certain aspects of procedural rationality, patients must still define their own ends for the HBCA to be successful. However, the opaque inclusion of others’ values and preferences regarding the desirability of those ends ultimately undermines the HBCA’s central claim to promote the patient’s own ends as defined by their values and preferences. As such, the presence of epistemic risk in screening programs undermines the ethical justification provided by the HBCA for using nudges, even accounting for a more flexible ecological view of rationality and autonomy.

Overall, both of the two proposed revisions to the HBCA - adopting a population-level perspective and incorporating an ecological conception of autonomy - fail to provide a satisfactory solution to the challenges posed by epistemic risk. The population-level approach abandons the core ethical justification of the original HBCA, respect for individual autonomy, and as such, requires a fundamentally different ethical framework. In contrast, the ecological approach aims to preserve this focus on individual autonomy, but it still fails to address the central issue: epistemic risk introduces non-patient values that may undermine an individual’s ability to define and pursue their own ends. As such, even these revised versions of the HBCA are undermined by the presence of epistemic risk in screening programs.

## Conclusion

In conclusion, the “help bad choosers argument” (HBCA) claims to provide an ethical justification for nudging in the context of screening, by suggesting that nudging can promote an individual’s own values and preferences. However, this argument is undermined by epistemic risk because others’ values and preferences are opaquely included in various points of the screening process. These exogenous values and preferences are directly relevant to patient decision-making in the context of screening. This undermines the HBCA due to (1) the possibility of conflicting values and preferences, (2) lack of transparency, (3) limited autonomy in opting out, and (4) manipulation. Thus, the HBCA is not consistent in its claim to promote patients’ ability to choose in line with their own values and preferences, and as a result, its ability to offer an ethical justification for nudging in screening is weakened.

The presence of epistemic risk in screening programs raises further questions about the influence of non-patient values on decision-making, highlighting the need for further examination of the role of nudging in promoting patient autonomy and informed consent. While not presenting a “knock-down” argument against the HBCA, this article raises a significant question as to whether nudging in screening really helps patients make decisions in line with their own preferences and values. Although epistemic risk has already been applied to analyzing healthcare practices, the argument here suggests that we should further examine the ways epistemic risk may also undermine bioethical arguments or justifications concerning such practices.

For example, the traditional model of informed consent in bioethics relies on the assumption that patients can make informed decisions based on the information provided to them. However, the concept of epistemic risk highlights an important limitation of this assumption, by introducing information that is relevant to patient decision-making that cannot be easily or clearly communicated. As such, bioethical arguments concerning informed consent that do not address epistemic risk may be neglecting an important ethical consideration. In general, considering epistemic risk adds to current questions about the extent to which patients can truly comprehend the complexities of their medical choices, especially in the context of rapidly evolving medical technologies and complex treatment options, and difficulties with the statistical basis of health-related information. Further research into how epistemic risk may influence the ethics of informed consent is needed to clarify these issues.

Aside from noting potential avenues for future research, it’s also important to emphasize that the argument I present here is very limited, and I am not arguing against the practice of screening itself. My argument specifically focuses on demonstrating how one particular type of rationale to support screening, namely, the “help bad choosers argument” (HBCA), is unsuccessful in meeting its own aims, at least without modification or additional support. This article does not aim to dictate whether screening should be carried out or avoided, but only to argue against the HBCA as an ethical justification for nudging in screening. Screening can still be justified on other grounds, as can nudging people into screening. For example, we could point directly to the health related benefits of screening, especially at the population level, as a justification for nudging. However, this kind of justification must be done with specific kinds of screening in mind, as it depends on the risk/benefit ratio of particular screening programs.

Further, this argument does not claim that the inclusion of *any* non-patient values necessarily undermines autonomy. Rather, it focuses specifically on the substantive values and preferences introduced by epistemic risk that may influence the choices patients make in the context of screening. While other values and preferences may be introduced through a variety of features of the patient’s environment, these are not necessarily the result of epistemic risk and, consequently, are outside the scope of this article.

Finally, with additional support or justification, the challenge I’ve raised for the HBCA may be lessened. For example, manipulation may be justified on other grounds, which would weaken the epistemic risk argument against the HBCA, by undercutting one of the reasons to believe that the inclusion of non-patient values and preferences raises an ethical problem. Similarly, changes in the organization of health care practices may help to improve transparency, which would strengthen the HBCA.^20^

In short, I argue that epistemic risk undermines the “help bad choosers argument,” and in doing so, undermines an important class of ethical justifications for nudging patients into screening programs. This, in turn, underscores the importance of a engingaging in further examination of epistemic risk in bioethical argumentation itself. Further exploration is needed in the ways that epistemic risk may influence various bioethical justifications of healthcare practices.

## Footnotes

1 Although it is a well-established healthcare practice, there is no single and unanimous definition of screening. See Juth and Munthe (2012), specifically Sect. 1.3, for philosophical discussion of the contested definitions of screening. See and Raffle and Gray (2019) specifically ch. 2, for further discussion. See also Speechley et al. (2017) for a systematic review of screening definitions.

2 It is important to note that ‘bad choosers’ are not just individuals who have some permanent or global choice-related deficiency. Rather, at certain points, we are all potentially “bad choosers.” As Hofmann and Stanak (2018) note, “at times, people are “bad choosers” as they may choose without being informed or be under strong influence from others” (p.1564). This fact, when combined with the various difficulties present in screening programs stemming from the statistical basis of the information presented to patients, means that many people, perhaps even most, are likely to fall into the ‘bad choosers’ category at some point (Hofmann 2020; Gigerenzer et al. 2007).

3 Note that in this discussion of epistemic risk, risk is understood as “exposure to harm (in the broadest sense) from acting in the face of uncertainty” (Biddle and Kukla 2017, p.218); compare with the variety of definitions of risk found in Hansson (2004).

4 For a more complete and comprehensive discussion of the concept and definition of epistemic risk, see Biddle and Kukla (2017), who also provide a useful mapping of various subtypes of epistemic risk.

5 In this article, “values and preferences” specifically refers to those directly relevant to the patient decision-making in the context of screening that arise from epistemic risk, rather than just any exogenous values and preferences that may be introduced.

6 Note that there are significant differences in how “bounded rationality” is understood. The basis of the understanding of bounded rationality described here is that rational decision theory yields the “correct” answers, and deviations from these answers are counted as errors or failures of rationality. However, there is significant debate over how, when, and to what extent deviations from decision theory should be properly counted as “biases” or “errors.” See Gigerenzer (2020) for discussion of these different understandings of bounded rationality. I rely on this understanding of bounded rationality because it is the understanding typically employed in nudging. It is not an endorsement of this particular understanding.

7 The basic idea of the HBCA is the same as the underlying idea behind informed consent. As one representative discussion notes, “Informed consent procedures are justified insofar as they protect autonomy: that is, insofar as they conduce to allowing agents to *shape their lives in accordance with their own values*.” (Levy 2014, p.298. Emphasis mine).

8 Although see Baldwin (2014) for discussion of how nudges that claim to promote an individual’s own conception of welfare often end up changing that conception (p. 846).

9 See Vugts et al. (2020) for an excellent empirical review of the conceptualizations of autonomy at work in the literature on the ethics of nudging.

10 See, e.g. Brooks (2013), Cohen (2013), Gold and Lichtenberg (2012), Baldwin (2014), Vugts et al. (2020), Conly (2013), Nys and Engelen (2017), and Levy (2014) for representative instances of the HBCA. Also see Hofmann and Stanak (2018) for a broad discussion of the HBCA in screening.

11 In contrast, note that Gigerenzer (2015) argues that nudging may increase screening uptake, but also offers evidence that it may reduce understanding (specifically, see p.362).

12 However, undermining the arguments in favor of nudging may also be relevant for the practice of health care.

13 For a more complete and comprehensive discussion of the concept and definition of epistemic risk, see Biddle and Kukla (2017), who also provide a useful mapping of various subtypes of epistemic risk.

14 It is important to note that Biddle distinguishes different kinds of epistemic risks arising in these different stages. See Biddle (2020) Sect. 4 and Sect. 5.1, particularly page 6.

15 As a result, this may also undermine the purely “deliberative” or “facilitative” versions of the HBCA, which do not focus on promoting patient values or preferences, but on strengthening their deliberative capacities (e.g. the “facilitative conception” of nudging by Saghai (2013). The values and preferences embedded in the screening process cannot be communicated, and thus, cannot be objects of deliberation and intentional reflection.

16 To be clear, there are distinct decision points in the screening process which may involve nudging and epistemic risk. Nudges can potentially occur at multiple stages, including, pre-screening (e.g. default scheduling to increase participation), after screening results (e.g. nudges encouraging follow-up testing), and after diagnosis (e.g. nudges toward treatment options). Epistemic risk and the inclusion of non-patient values are relevant to nudges that may occur at each of these decision points, as the values involved at each stage are carried forward into any subsequent nudges based on the results of that stage. My claim is not that a single nudge spans all these points, but rather that the entire process up to the point patients are nudged incorporates substantive value judgments from epistemic risk, ultimately undermining the claim that nudges promote only the patient’s own values.

17 See Biddle (2020) for further discussion of how such values may be disclosed.

18 I am grateful to two anonymous reviewers who suggested I examine these two possibilities for revising the HBCA.

19 I’ve focused on issues related to autonomy here, but the P-HBCA faces other potential problems. For example, it must address the question of when it is justifiable to impose costs on some individuals to benefit others, as the individuals harmed by screening (e.g. from overdiagnosis) are typically different individuals from those who benefit. Additionally, the P-HBCA raises concerns about whose values are being promoted, as average group preferences may not adequately represent the diversity of individual preferences.

20 See Biddle (2020) for discussion of changing the organization of health care to better address epistemic risk.

## References

[CR1] Baldwin, R. 2014. From regulation to behaviour change: giving nudge the third degree. *The Modern Law Review* 77(6): 831–857. 10.1111/1468-2230.12094

[CR2] Biddle, J. B. 2007. Lessons from the Vioxx debacle: what the privatization of Science can teach us about Social Epistemology. *Social Epistemology* 21(1): 21–u9. 10.1080/02691720601125472

[CR3] Biddle, J. B. 2016. Inductive risk, epistemic risk, and overdiagnosis of disease. *Perspectives on Science* 24(2): 192–205. 10.1162/POSC_a_00200

[CR4] Biddle, J. B. 2020. Epistemic risks in cancer screening: implications for ethics and policy. *Studies in History and Philosophy of Science Part C: Studies in History and Philosophy of Biological and Biomedical Sciences* 79: 101200. 10.1016/j.shpsc.2019.101200 31387780

[CR5] Biddle, J. B., and R. Kukla. 2017. The geography of epistemic risk. In Elliot, H., and Richards, T., (eds.) *Exploring inductive risk: Case studies of values in science*, 215–237.

[CR6] Blumenthal-Barby, J. S., and H. Burroughs. 2012. Seeking better health care outcomes: the ethics of using the nudge. *The American Journal of Bioethics* 12(2): 1–10. 10.1080/15265161.2011.634481 22304506

[CR7] Brooks, T. 2013. Should we nudge informed consent? *The American Journal of Bioethics* 13(6): 22–23. 10.1080/15265161.2013.781710 23641841

[CR8] Coggon, J. 2020. Smoke free? Public Health Policy, Coercive Paternalism, and the Ethics of Long-Game Regulation. *Journal of Law and Society* 47(1): 121–148. 10.1111/jols.12213

[CR9] Cohen, S. 2013. Nudging and informed consent. *The American Journal of Bioethics* 13(6): 3–11. 10.1080/15265161.2013.781704 23641835

[CR10] Conly, S. 2013. Coercive paternalism in health care: against freedom of choice. *Public Health Ethics* 6(3): 241–245. 10.1093/phe/pht025. 10.1093/phe/pht025

[CR11] Damhus, C. S., Byskov Petersen, G. Ploug, T., and J. Brodersen. 2018. Informed or misinformed choice? Framing effects in a national information pamphlet on colorectal cancer screening. *Health Risk & Society* 20(5–6): 241–258. 10.1080/13698575.2018.1499877

[CR12] Douglas, C., and E. Proudfoot. 2013. Nudging and the complicated real life of informed consent. *The American Journal of Bioethics* 13(6): 16–17. 10.1080/15265161.2013.781716 23641838

[CR13] Ebell, M. H., T. N. Thai, and K. J. Royalty. 2018. Cancer screening recommendations: an international comparison of high income countries. *Public Health Reviews* 39(1): 1–19. 29507820 10.1186/s40985-018-0080-0 PMC5833039

[CR14] Epstein, W. N. 2017. Nudging patient decision-making. *Washington Law Review* 92(3): 1255–1315.

[CR16] Gigerenzer, G. 2015. On the supposed evidence for libertarian paternalism. *Review of Philosophy and Psychology* 6: 361–383. 26213590 10.1007/s13164-015-0248-1 PMC4512281

[CR17] Gigerenzer, G. 2020. What is bounded rationality? In *Routledge handbook of bounded rationality*, 55–69. Routledge.

[CR15] Gigerenzer, G., W. Gaissmaier, E. Kurz-Milcke, L. M. Schwartz, and S. Woloshin. 2007. Helping doctors and patients make sense of health statistics. *Psychological Science in the Public Interest* 8(2): 53–96. 26161749 10.1111/j.1539-6053.2008.00033.x

[CR18] Gold, A., and P. Lichtenberg. 2012. Don’t call me nudge: the ethical obligation to use effective interventions to promote public health. *The American Journal of Bioethics* 12(2): 18–20. 10.1080/15265161.2011.634486 22304511

[CR19] Gorin, M., S. Joffe, N. Dickert, and S. Halpern. 2017. Justifying clinical nudges. *Hastings Center Report* 47(2): 32–38. 10.1002/hast.688. 10.1002/hast.688 28301695

[CR20] Hansson, S. O. 2004. Philosophical perspectives on risk. *Techné: Research in Philosophy and Technology* 8(1): 10–35.

[CR21] Hausman, D. M., and B. Welch. 2010. Debate: to nudge or not to nudge*. *Journal of Political Philosophy* 18(1): 123–136. 10.1111/j.1467-9760.2009.00351.x. 10.1111/j.1467-9760.2009.00351.x

[CR22] Hofmann, B. 2016. Medicalization and overdiagnosis: different but alike. *Medicine Health Care and Philosophy* 19: 253–264. 26912187 10.1007/s11019-016-9693-6

[CR23] Hofmann, B. 2018. Looking for trouble? Diagnostics expanding disease and producing patients. *Journal of Evaluation in Clinical Practice* 24(5): 978–982. 29790242 10.1111/jep.12941

[CR24] Hofmann, B. 2020. Informing about mammographic screening: ethical challenges and suggested solutions. *Bioethics* 34(5): 483–492. 31633832 10.1111/bioe.12676

[CR25] Hofmann, B., and M. Stanak. 2018. Nudging in screening: literature review and ethical guidance. *Patient Education and Counseling* 101(9): 1561–1569. 29657111 10.1016/j.pec.2018.03.021

[CR26] Juth, N., and C. Munthe. 2012. *The ethics of screening in health care and medicine: serving society or serving the patient?*. Dordrecht: Springer.

[CR27] Kahneman, D., S. P. Slovic, P. Slovic, and A. Tversky. eds. 1982. *Judgment under uncertainty: Heuristics and biases*. Cambridge University Press. 10.1126/science.185.4157.1124 17835457

[CR28] Kuyer, P., and B. Gordijn. 2023. Nudge in perspective: a systematic literature review on the ethical issues with nudging. *Rationality and Society*, 10434631231155005.

[CR29] Levy, N. 2014. Forced to be free? Increasing patient autonomy by constraining it. *Journal of Medical Ethics* 40(5): 293–300. 22318413 10.1136/medethics-2011-100207 PMC3995287

[CR43] Moynihan, R., Doust, J., & Henry, D. 2012. Preventing overdiagnosis: How to stop harming the healthy. *BMJ : British Medical Journal*, 344, e3502. https://doi.org/10.1136/bmj.e3502 10.1136/bmj.e3502 22645185

[CR30] Munoz, R., M. Fox, M. Gomez, and S. Gelfand. 2015. Evidence-based nudging: best practices in informed consent. *The American Journal of Bioethics* 15(10): 43–45. 10.1080/15265161.2015.1074311 26479102

[CR31] Nys, T. R., and B. Engelen. 2017. Judging nudging: answering the manipulation objection. *Political Studies* 65(1): 199–214. 10.1177/0032321716629487

[CR32] Olson, B., B. Gribble, J. Dias, C. Curryer, K. Vo, P. Kowal, and J. Byles. 2016. Cervical cancer screening programs and guidelines in low-and middle-income countries. *International Journal of Gynecology & Obstetrics* 134(3): 239–246. 27350227 10.1016/j.ijgo.2016.03.011

[CR33] Plutynski, A. 2017. Safe or sorry? Cancer screening and inductive risk. In *Exploring inductive risk: Case studies of values in Science*, 149–170. Oxford University Press.

[CR34] Raffle, A. E., and J. M. Gray. 2019. *Screening: evidence and practice*. USA: Oxford University Press.

[CR35] Saghai, Y. 2013. Salvaging the concept of nudge. *Journal of Medical Ethics* 39(8): 487–493. 23427215 10.1136/medethics-2012-100727

[CR36] Schmidt, A. T. 2019. Getting real on rationality—behavioral science, nudging, and public policy. *Ethics* 129(4): 511–543. 10.1086/702970

[CR37] Schmidt, A. T., and B. Engelen. 2020. The ethics of nudging: an overview. *Philosophy Compass* 15(4): e12658. 10.1111/phc3.12658

[CR38] Simkulet, W. 2017. Nudging, informed consent and bullshit. *Journal of Medical Ethics* 44(8): 536–542. 29151058 10.1136/medethics-2017-104480

[CR39] Speechley, M., A. Kunnilathu, E. Aluckal, M. S. Balakrishna, B. Mathew, and E. K. George. 2017. Screening in public health and clinical care: similarities and differences in definitions, types, and aims–a systematic review. *Journal of Clinical and Diagnostic Research: JCDR* 11(3): LE01. 10.7860/JCDR/2017/24811.9419 PMC5427344 28511418

[CR40] Thaler, R. H., and C. R. Sunstein. 2009. *Nudge: improving decisions about health, wealth, and happiness*. Penguin.

[CR41] Vugts, A., M. van Den Hoven, E. De Vet, and M. Verweij. 2020. How autonomy is understood in discussions on the ethics of nudging. *Behavioural Public Policy* 4(1): 108–123. 10.1017/bpp.2018.5

[CR44] Welch, H. G., Schwartz, L., & Woloshin, S. 2011. *Overdiagnosed: Making people sick in the pursuit of health*. Boston: Beacon.

[CR42] Waldron, J. 2014. It’s all for your own good. *The New York Review of Books*, (October 9, 2014). http://www.nybooks.com/articles/archives/2014/oct/09/cass-sunstein-its-all-your-own-good/.

